# Preoperative Portal Vein Embolization for Liver Resection: An updated meta-analysis

**DOI:** 10.7150/jca.50371

**Published:** 2021-01-21

**Authors:** Yu Huang, Wenhao Ge, Yang Kong, Yuan Ding, Bingqiang Gao, Xiaohui Qian, Weilin Wang

**Affiliations:** 1 Department of Hepatobiliary and Pancreatic Surgery, The Second Affiliated Hospital, Zhejiang University School of Medicine, Hangzhou, Zhejiang 310009.; 2 Key Laboratory of Precision Diagnosis and Treatment for Hepatobiliary and Pancreatic Tumor of Zhejiang Province, Hangzhou, Zhejiang 310009.; 3 Research Center of Diagnosis and Treatment Technology for Hepatocellular Carcinoma of Zhejiang Province, Hangzhou, Zhejiang 310009.; 4 Clinical Medicine Innovation Center of Precision Diagnosis and Treatment for Hepatobiliary and Pancreatic Disease of Zhejiang University, Hangzhou, Zhejiang 310009.; 5 Clinical Research Center of Hepatobiliary and Pancreatic Diseases of Zhejiang Province, Hangzhou, Zhejiang 310009.

**Keywords:** portal vein embolization, liver tumor, future liver remnant, liver regeneration, liver resection

## Abstract

**Background:** Portal vein embolization (PVE) is performed before major liver resection to increase liver volume remnant, controversy remains on the adverse effect of PVE on liver tumor patients. The current study highlighted the effect of PVE on the degree of hypertrophy of future liver remnant (FLR) and summarized PVE-related complications, aiming to provide a guideline for surgeons.

**Methods:** A search of current published studies on PVE was performed. Meta-analysis was conducted to assess the effect of PVE on hypertrophy of FLR and summarized PVE-related complications.

**Results:** 26 studies including 2335 patients were enrolled in the meta-analysis. All enrolled studies reported data regarding FLR hypertrophy rate, pooled effect size (ES) for FLR hypertrophy rate using a fixed-effect model was 0.105 (95%CI: 0.094-0.117, p=0.000), indicating PVE is favored in inducing FLR hypertrophy. Metatrim method indicated no obvious evidence of publication bias in the present meta-analysis. 247 (10.6%) patients exhibited PVE-related complications, receiving expectant treatment without affecting planned liver resection. Total 1782 patients (76%) underwent a subsequent liver resection after PVE, which is an encouraging result comparing with traditional resection rate in liver tumor patients.

**Conclusions:** PVE is a safe and effective procedure with a low occurrence of related complications for inducing sufficient hypertrophy of FLR in liver tumor patients, which could elevate the resection rate of liver tumor patients. Careful patient cohort selection is crucial to avoid overuse of PVE in technically resectable patients. Further multiple central clinical trials are conducive to select optimal patient cohorts and provide a guideline for surgeons.

## Introduction

Liver resection remains the gold standard treatment offering both potential cure and long-term survival to patients with either primary or secondary liver tumors 1,2. The aim of resection is to offer a curative effect with reservation of a sufficient future liver remnant (FLR) to maintain basic liver function at the same time in patients with liver tumors 3,4. Unfortunately, at the time of diagnosis, only <25% of patients are suitable for surgical resection 5. Meanwhile, the resection rate for the liver tumor is just 20%-30% in patients with normal livers even reduced in patients with cirrhotic liver. For up to 45% of liver tumor patients, an extended liver resection is imperative to achieve absolute clear resection margins 6. One of the reasons for aforementioned unresectability is that the remnant liver is insufficient to support postoperative liver function 7. Postoperative liver failure is still one of the main causes of death following major liver resection, ranging from 0 to 30%, with insufficient FLR being a limiting factor 4. In literature, postoperative liver failure is directly associated with the volume of liver remnant 8. To ensure sufficient liver remnant volume after liver resection, several strategies, including portal vein embolization (PVE), portal vein ligation (PVL), associating liver partition with portal vein ligation for staged hepatectomy (ALPPS) procedure, and selective internal radiation therapy (SIRT), have been recently employed in inducing hypertrophy of FLR 9,10. Within them, ALPPS procedure has been demonstrated to achieve the greatest increase rate of FLR recently 11. However, PVE has been sometimes recognized as a more ideal method for inducing a comparable increase rate of FLR with ALPPS procedure as well as its lower morbidity and mortality than ALPPS, which is widely accepted by the majority of centers before major liver resection 12,13.

Portal vein embolization, of which the basic principle involved in occluding a branch of portal venous flow to the liver segments that are planned to resect, subsequently results in ipsilateral hepatic atrophy and compensatory contralateral hypertrophy, was first described by Kinoshita in a hepatocellular carcinoma (HCC) patient in 1986 12. Since then, various studies have reported the efficacy of PVE in inducing compensatory hypertrophy of FLR in preparation for liver resection 14-17. Currently, PVE is usually performed as a routine procedure before any extended liver resection to increase remnant liver volume 18. Although, many clinical studies have been published on hypertrophy of the FRL in small and large patient cohorts. Controversy remains on the potential adverse effect of PVE on tumor growth. Some studies suggested that PVE also stimulates the growth of liver tumor that is still present within the regenerating liver, regardless of embolized lobe or the non-embolized lobe. Disease progression secondary to PVE may affect surgical strategies and patient outcomes 19,20. Meanwhile, concerns are also raised as to whether PVE only induces volume change rather than functional gain 21.

Two meta-analyses have been published on the effect of PVE in major liver resection. The first by Abulkhir et al. in 2008 reviewed different techniques (percutaneous transhepatic and transileocolic) of PVE and concluded that PVE is an effective procedure in inducing liver regeneration to prevent postoperative liver failure 21. Another by Lienden et al. demonstrated that PVE has a high technical and clinical success rate and liver cirrhosis has a negative effect on the hypertrophy induced by PVE 8. However, there is still no authoritative literature systematically summarized the advantages and adverse effects of PVE. In our present meta-analysis, we mainly highlighted the effect of PVE on the degree of hypertrophy of FLR and summarized PVE-related complications, aiming to provide a guideline for surgeons to make an accurate decision.

## Materials and Methods

### Search strategy and study selection

**Figure 1:**
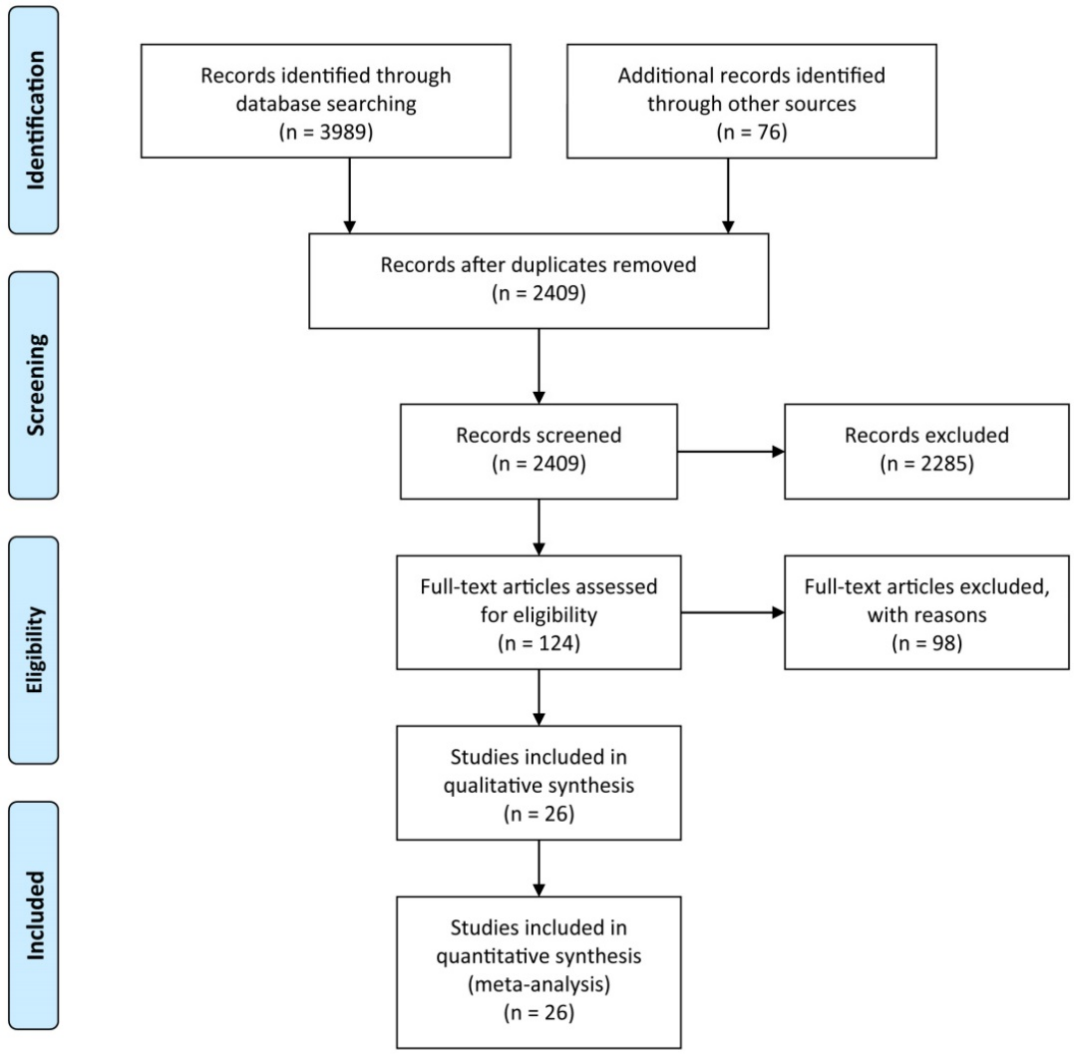
The Preferred Reporting Items for Systematic Reviews and Meta-Analyses (PRISMA) flow diagram for search and selection processes of the meta-analysis.

A systematic search of the available published studies on portal vein embolization was conducted in Pubmed, Embase, Medline, PMC, Web of Science, and Cochrane database. Two researchers (Y.H. and W.G.) independently searched publications from 1990 to March 2020 using the following “Mesh Terms”: “portal vein embolization”, “liver resection”, and “liver tumor”. The “related article” function was also used to broaden the search. All abstracts, studies, and citations retrieved were reviewed, including references of these articles. The final selection of the articles was made in consensus by all authors. The detail of literature search strategies is illustrated in Figure 1.

### Eligibility criteria

All full text articles were enrolled if they were composed of information on patient characteristics, indications for PVE, techniques, and materials of PVE, the hypertrophy rate of FLR, the successful rate of resection and complications after PVE. Newcastle-Ottawa Quality Assessment Scale (NOS) bias risk tool was used to assess the methodological quality of enrolled studies, and those with a score ≥7 were considered eligible and enrolled in our study. We then extracted the aforementioned clinical parameters from enrolled studies.

### Exclusion criteria

We excluded studies if they are reviews, case reports, animal studies, non-English publications, and repetitive publications in different databases. Studies that didn't record patient characteristics, FLR before and after PVE or the hypertrophy rate of FLR, and complications after PVE were also excluded. We also excluded the studies in which appropriate data could not extract from the results.

### Statistical analysis

The meta-analysis was performed according to recommendations from the Cochrane Collaboration and the Quality of Reporting of Meta-analyses (QUORUM) guidelines. Single-rate meta-analysis was performed using Stata 12.0 software (Stata Corporation, College Station, TX, USA). The combined effect size (ES) of FLR hypertrophy rate was examined. Combined ES more than 0 favored in the efficacy of PVE and the point estimate of ES was considered to be statistically significant at *P* < 0.05 level if the 95% confidence interval didn't include the value 0. Heterogeneity among the studies was tested using the *p* value of Q test and *I*^2^ test. When *p* > 0.1 and* I*^2^ ≤50%, a fixed-effect model was used, otherwise a random effect model was selected. A further sensitivity analysis was performed to detect the heterogeneity. Funnel plot, as well as metatrim method, was used to detect the publication bias. *P* < 0.05 was considered as statistically significant.

## Results

### Research selection and quality assessment

Based on the aforementioned search strategies, 4065 publications including related articles were searched from the online database. After removing repetitive publications, a total of 2409 records remained. Then, 2285 publications were excluded by screening the titles and abstracts, and 98 of the remaining 124 articles were deleted for various reasons. At last, 26 publications with an NOS score ≥7, including 2335 patients were enrolled in the present meta-analysis (Figure 1). The characteristics of the enrolled studies and clinical parameters of patients in these studies were summarized in Tables 1-3.

### FLR hypertrophy rate

**Figure 2:**
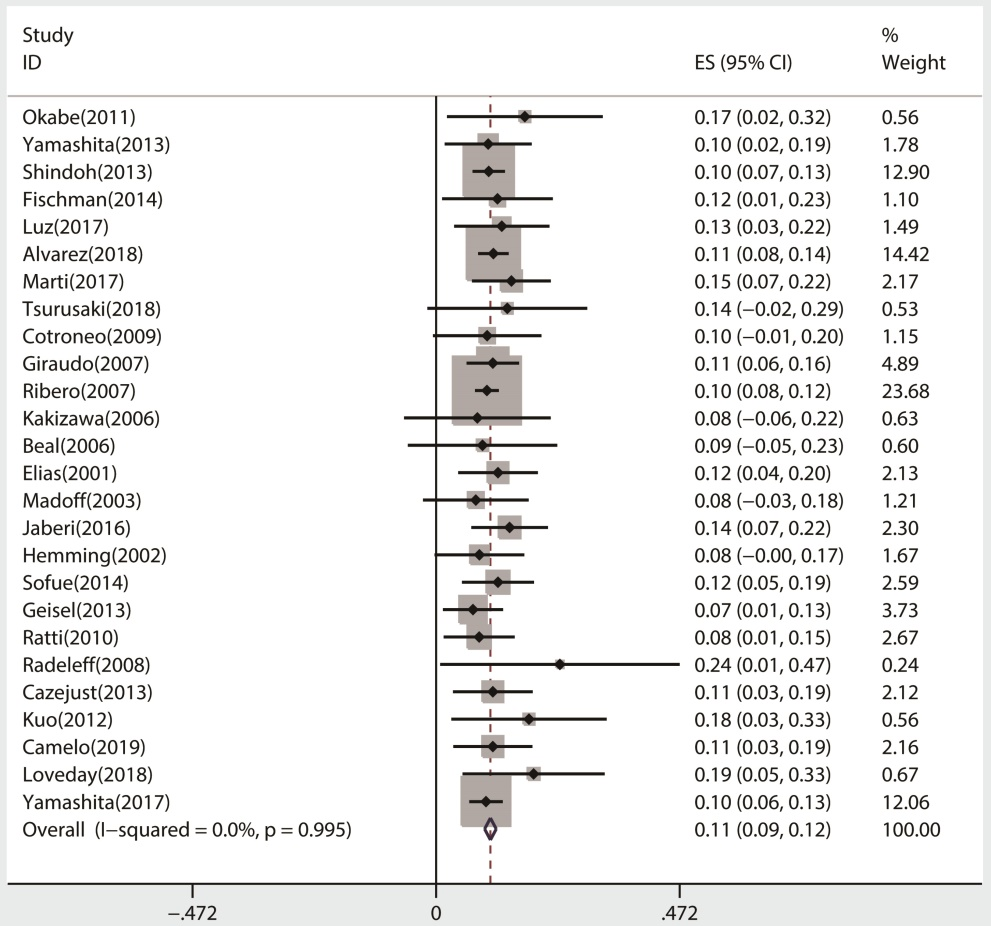
Meta-analysis of effect size (ES) of hypertrophy rate in future liver remnant after PVE.

**Figure 3:**
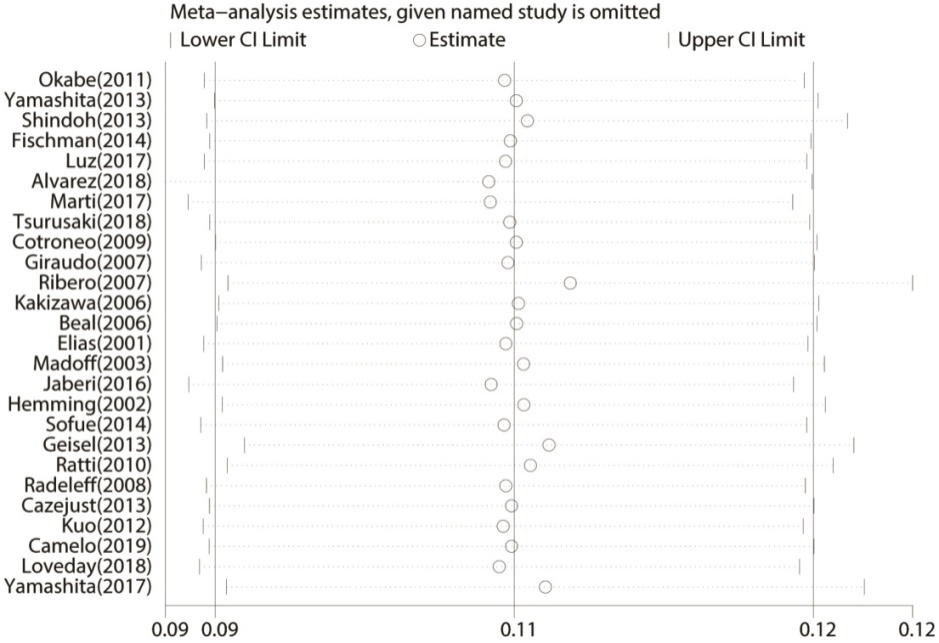
Sensitivity analysis of the meta-analysis.

All 26 studies reported data regarding FLR hypertrophy rate, pooled ES for FLR hypertrophy rate using a fixed-effect model was 0.105 (95%CI: 0.094-0.117, p=0.000), indicating PVE is favored in inducing FLR hypertrophy. Additionally, the sensitivity analysis demonstrated that there is no study that greatly interfered with the results of the present meta-analysis, suggesting no proof of heterogeneity among the enrolled studies (*p* value of Q test=0.995, *I*^2^=0%) (Figures 2 & 3).

### Publication bias

**Figure 4:**
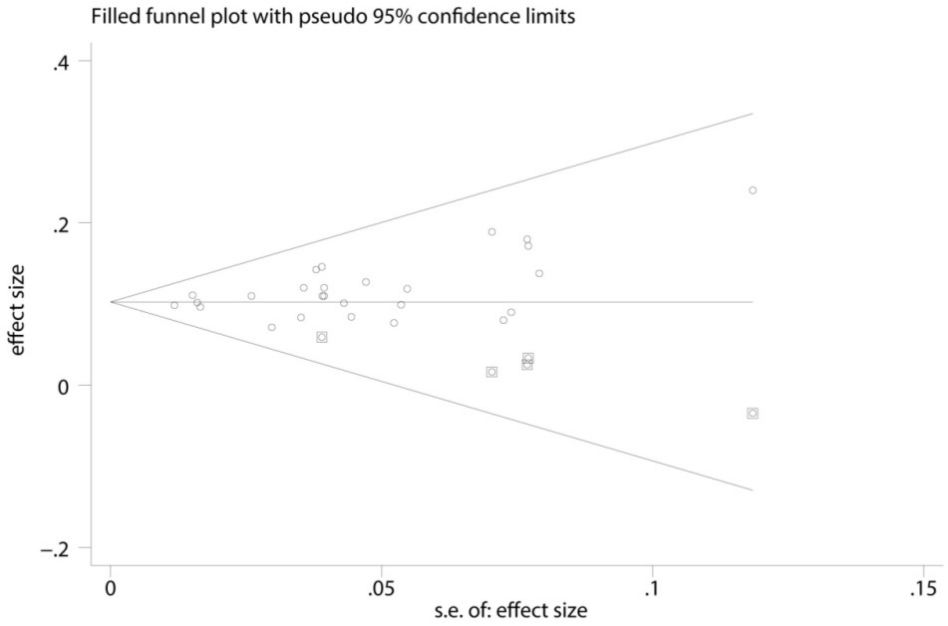
Funnel plot, as well as metatrim method, assess the publication bias of the meta-analysis.

A funnel plot, as well as metatrim method was used to detected publication bias. Before metatrim, pooled ES for FLR hypertrophy rate was 0.105 (95%CI: 0.094-0.117, p=0.000). After metatrim, 5 studies were added into the meta-analysis, and pooled ES for FLR hypertrophy rate was 0.103 (95%CI: 0.092-0.114, p=0.000). Results before and after metatrim are stable and are both statistically significant, which means publication bias is negligible in the present study (Figure 4).

### PVE-related complications

Although almost every enrolled study reported the complications, 247 (10.6%) patients exhibited PVE-related complications, of which abdominal pain, fever, and coil displacement are most frequently seen. The overall occurrence rate of complications is infrequent after PVE, and there was no mortality directly associated with PVE. All patients with complications received expectant treatment without affecting subsequent liver resection (Table 4).

### Liver resection after PVE

In the present study, 1782 patients (76%) underwent a subsequent liver resection after PVE, which is an encouraging result comparing with the traditional resection rate in liver tumor patients. The average interval between PVE and surgery was 38.9 days, resembling the results ever reported. 553 patients (24%) failed to undergo operations because of insufficient hypertrophy, local tumor progression, extrahepatic tumor spread and other complications (Table 3).

## Discussion

Preoperative PVE has been performed clinically to induce hypertrophy of the contralateral lobe and avoid postoperative liver failure resulted from insufficient remnant liver after resection. The basic principle of PVE is occluding a branch of portal venous flow to the liver segments that are planned to resect, resulting in ipsilateral hepatic atrophy and compensatory contralateral hypertrophy 18. However, the exact molecular mechanism leading to atrophy of the embolized lobe and hypertrophy of the FLR is still unknown. Recent studies showed that hepatic growth factor (HGF) and transforming growth factor (TGF)-α and -β may play vital roles in contributing to the hypertrophy of the non-embolized lobe 22.

As for the indications, PVE is initially used to increase the resection rate in HCC patients 12. Over the past two decades, the indications of PVE also include nearly all primary and secondary liver tumors with insufficient FLR before major liver resection 23-29. Ribero et al. showed a small FLR is strongly associated with postoperative hepatic dysfunction 9. Hence, the majority of centers use an FLR volume ratio of 25%-30% of the original liver volume as a threshold to select appropriate patients with normal liver function. Nevertheless, most liver tumor patients are usually with the infection of hepatic virus, the history of chemotherapy, liver cirrhosis or fibrosis, and other factors inducing liver dysfunction. A threshold of 35%-45% is preferred by most centers as a minimum FLR volume rate 3,23. Some Japanese researchers also advocate to select appropriate patients for PVE by the method of indocyanine green (ICG) plasma disappearance or retention rate test at 15 min, which is beneficial to estimate preoperative remnant liver function 30. Recent researches reported quantitative liver function tests, such as ^99^Tc-labelled mebrofenin hepatobiliary scintigraphy HBS and ^99^Tc-galactosyl-human serum albumin (GSA) scintigraphy, are conducive to select appropriate patients for PVE 31.

Several mature techniques for PVE have been introduced, including transileocolic portal vein embolization (TIPE), the percutaneous transhepatic ipsilateral or contralateral PVE technique (PTPE), and intraoperative portal branch embolization 32-34. It is demonstrated that a greater increase in FRL in PTPE than in surgical TIPE, as well as no difference in the occurrence of major complications 6. With the advancement of radiological intervention, PTPE becomes the standard technique for PVE with a satisfactory success rate. PTPE can be performed by an ipsilateral or contralateral approach. The ipsilateral approach is preferred by the majority of centers for its advantage of avoiding puncturing the FLR tissue and easier to access to segment Ⅳ, though technically more difficult 35.

Many available commercially embolization materials have been applied for PVE. Polyvinyl alcohol (PVA) particles and N-butyl-cyanoacrylate with coils are mostly used 8,36. In our meta-analysis, we summarized 26 studies and concluded that apart from both of them, absolute ethanol, microspheres, and gelatin powder are also widely accepted in the majority of centers 37-40. (Table 3) N-butyl-cyanoacrylate induces severe inflammatory reaction, usually resulting in technical difficulty in surgical resection 14,41. Gelatin powder is absorbable, producing only transient embolization with the possibility of vascular recanalization 42. Absolute ethanol has been showed to induce peripheral parenchyma fibrosis and necrosis, and severe abdominal pain sometimes, though producing effective hypertrophy of FLR 43. PVA particles are easily available and provide persistent occlusion of portal branched with acceptable side effects. Hence, PVA is recommended to apply alone or with other materials in the majority of centers 44,45. In general, large clinical studies comparing different embolization materials are still necessary to seek the optimal materials.

All patients underwent volumetric assessment by means of CT imaging before PVE and surgery 46. There is no consensus on the most appropriate waiting time between PVE and surgery. It has been showed that the average interval from PVE to liver resection was 29 days 21. In our study, the majority of the enrolled studies reported interval between PVE and liver resection, the average interval was 38.9 days (Table 3), which is similar to the results ever reported. To our knowledge, a longer time interval after PVE allows greater growth of FRL. Nevertheless, there is the issue put forward by some surgeons that tumor growth is simultaneously induced by PVE. Accumulating studies demonstrated that tumor progression after PVE is possible in both embolized and non-embolized lobe 19,47. Additionally, controversy remains as to whether PVE only induces volume change rather than functional gain 21. In consideration of disease progression after PVE may affect surgical strategies and patient outcomes, more multiple central clinical trials are imperative to come to a consensus on the optimal interval between PVE and liver resection.

Apart from limiting time between PVE and liver resection, post-PVE chemotherapy, or sequential transarterial chemoembolization (TACE) is also recommended to restrict tumor progression by some centers 8. Beal et al. demonstrated a reduction in tumor size in patients who received chemotherapy after PVE. However, the attendant problem is that less hypertrophy of FRL is observed in patients with a history of chemotherapy 48. Other studies also showed no significant difference in hypertrophy rate or complications in patients with chemotherapy post PVE 49. Due to the limited number of current studies and their heterogeneity, more researches are needed to evaluate the effect of chemotherapy on the PVE receptor.

Either the overall technical success (99.3%) or clinical success rate (96.1%) of PVE is extremely high as reported. Patients who experienced failure for the first time also possess the second chance to achieve a successful embolization, which made PVE a safe and effective technique for patients 8. Although various PVE-related complications have been reported, complications infrequently occurred after PVE and there was no mortality directly associated with PVE. In our present study, 247 (10.6%) patients exhibited PVE-related complications, of which abdominal pain, fever, and coil displacement are most frequently seen (Table 4). All patients with complications received expectant treatment without affecting subsequent liver resection. In our present meta-analysis, 553 patients (24%) failed to undergo a liver resection because of insufficient hypertrophy, local tumor progression, extrahepatic tumor spread, and other PVE-related complications (Table 3). However, comparing with traditional resection rate in liver tumor patients, more patients benefit from PVE and have access to resection with a reduced occurrence of postoperative complications.

## Conclusion

Although as one of the emerging methods inducing hypertrophy of FLR, PVE has been expertly used during recent years with an acceptable adverse effect to make more patients able to achieve major liver resection with a high rate of success, which is recommendable for any patients with a small future liver remnant volume when considering liver resection. Our previous teamwork reported that PVE prior to hepatectomy may promote FLR compensatory hypertrophy and an increase in the resectability of primary liver cancer, which could be considered as an independent patient cohort to validate our findings and conclusions in our present meta-analysis 50,51. The ipsilateral approach is preferred and PVA particles are usually the first choice for PVE. More multiple central clinical trials are needed to determine whether it is necessary to conduct post-PVE chemotherapy and when is the appropriate time to perform the resection. PVE-related complications are infrequently seen and timely expectant treatment is beneficial for patients without affecting subsequent liver resection.

## Superiority

To date, this is the first meta-analysis that directly highlighted the degree of hypertrophy of FLR by PVE procedure. Due to the greatest patient cohort and rational analysis method in the present meta-analysis, the statistical power of this meta-analysis and the integrity of the summary were better than any individual research published so far. Additionally, this meta-analysis contributes new convincing information to previous literature, which may provide a promising guideline for other researchers.

## Limitations

Meta-analysis has an intrinsic bias introduced by the selection and location of studies. Meanwhile, researchers preferred to report positive findings and studies with significant differences are easy to be published, which may induce publish bias. In our present meta-analysis, most of the enrolled studies are retrospective, of which a long study period may introduce potential confounders. In addition, although a large patient cohort is included, the quality of enrolled studies is uneven, which may result in bias in our result. Hence, more high quality randomized, clinical trials are conducive to select the most appropriate patient cohorts and evaluate the effect of PVE.

## Figures and Tables

**Table 1:** Description of the 26 studies enrolled in the meta-analysis

Author	Year	Country	Inclusion period	Age	No. of patients	Resection patients	Interval between PVE and surgery	NOS score
Okabe 46	2011	Japan	1999-2009	58.8 (40-78)	24	19	28 (19-63)	7
Yamashita 2	2013	Japan	1996-2009	61 (35-81)	64	49	NR	7
Shindoh 6	2013	America	1995-2012	58 (24-86)	358	282	32 (5-385)	8
Fischman 37	2014	America	2011-2013	59.9 (34-76)	35	27	41.6 (26-78)	9
Luz 41	2017	Brazil	NR	56.5 (27-86)	50	31	NR	7
Alvarez 23	2018	France	1993-2015	60 (24-86)	431	287	50 (35-69.5)	7
Marti 24	2017	America	2006-2014	61 (51.8-68)	82	69	37 (20-135)	8
Tsurusaki 32	2018	Japan	2010-2016	69.5 (45-86)	19	19	NR	7
Cotroneo 25	2009	Italy	NR	66.2 (54-77)	31	24	NR	7
Giraudo 33	2007	France	1997-2006	64 (44-88)	145	114	NR	7
Ribero 9	2007	America	1995-2006	60 (36-78)	112	78	NR	7
Kakizawa 42	2006	Japan	2001-2005	65 (35-81)	14	11	22 (14-37)	8
Beal 48	2006	British	1999-2002	65 (52-74)	15	8	NR	7
Elias 16	2001	France	1987-2000	NR	68	60	30 (24-65)	7
Madoff 36	2003	America	1998-2001	59 (29-77)	26	16	NR	7
Jaberi 44	2016	Canada	2008-2013	61.2 (38-84)	85	60	NR	8
Hemming 26	2002	America	1996-2002	61 (31-82)	39	31	NR	7
Sofue 43	2014	Japan	2007-2011	68 (45-82)	83	69	25 (14-55)	7
Geisel 38	2013	Germany	2011-2012	NR	75	70	NR	7
Ratti 27	2010	Italy	2006-2009	63 (37-82)	62	56	35 (13-57)	8
Radeleff 39	2008	Germany	2001-2006	55 (31-68)	15	11	49 (34-72)	9
Cazejust 40	2013	France	2009-2013	63 (38-80)	63	49	34 (28-49)	8
Kuo 17	2012	Australia	1998-2007	60 (46-78)	25	19	36 (17-180)	7
Camelo 45	2019	Portugal	2013-2017	64 (42-84)	64	44	NR	7
Loveday 28	2018	America	2008-2015	61.8 (39-80)	31	23	8 (4-58)	9
Yamashita 29	2017	Japan	1995-2013	63 (22-81)	319	256	NR	7

**Abbreviation:** NOS: Newcastle-Ottawa Quality Assessment Scale Score; NR: not reported.

**Table 2:** PVE indications

Author	PVE indications
Okabe 46	ICG≤10% and FLR<35% or 10%<ICG<20% and FLR<60%
Yamashita 2	FLR≤40%
Shindoh 6	FLR≤20% in patients with normal liver or FLR≤30% in patients with liver fibrosis
Fischman 37	FLR≤20% in patients with normal liver or FLR≤30% in patients with history of chemotherapy or FLR≤40% in patients with liver fibrosis
Luz 41	FLR≤25% in patients with normal liver or FLR≤40% in patients with liver fibrosis
Alvarez 23	FLR≤30% in patients with normal liver or FLR≤40% in patients with liver fibrosis
Marti 24	FLR≤40%
Tsurusaki 32	NR
Cotroneo 25	FLR≤25%
Giraudo 33	FLR≤30% in patients with normal liver or FLR≤40% in patients with liver fibrosis
Ribero 9	FLR≤20% in patients with normal liver or FLR≤30% in patients with history of chemotherapy or FLR≤40% in patients with liver fibrosis
Kakizawa 42	NR
Beal 48	NR
Elias 16	FLR≤30% in patients with normal liver or FLR≤40% in patients with history of chemotherapy
Madoff 36	FLR≤25%
Jaberi 44	FLR<30% in patients with normal liver or FLR<40% in patients with history of chemotherapy
Hemming 26	FLR≤25% in patients with normal liver or FLR≤40% in patients with liver fibrosis
Sofue 43	ICG<15% and FLR<40%
Geisel 38	FLR≤25% in patients with normal liver or FLR≤40% in patients with liver fibrosis
Ratti 27	FLR<30% in patients with normal liver or FLR<40% in patients with history of chemotherapy
Radeleff 39	FLR≤25% in patients with normal liver or FLR≤45% in patients with liver fibrosis
Cazejust 40	FLR≤25% in patients with normal liver or FLR≤30% in patients with history of chemotherapy or FLR≤40% in patients with liver fibrosis
Kuo 17	NR
Camelo 45	NR
Loveday 28	FLR≤40%
Yamashita 29	ICG<10% and FLR≤40% or 10%<ICG≤20% and FLR<50%

**Abbreviation:** PVE: portal vein embolization; ICG: indocyanine green; FLR: future liver remnant; NR: not reported.

**Table 3:** Baseline characteristic of patients in the enrolled studies

Details	No. (%)
Total no. patients	2335
Age (year)	61±14
Pathology	
HCC 6,9,17,23,24,25,36,27,28,29,32,33,36,37,38,39,40,43,44,45,46	528 (23)
CHC 6,9,17,23,25,27,29,32,33,36,37,38,39,40,41,42,43,44	558 (24)
CLM 2,6,9,16,17,23,25,26,27,29,32,33,36,37,38,39,40,41,42,43,44,45,48	1045 (45)
Others 6,9,23,25,26,27,32,33,36,38,39,40,41,42,43,44,45	204 (8)
Embolization materials	
Ethanolamine oleate iopamidol 46	24 (1)
Gelatinpowder+thrombin	319 (14)
+diatrizoate sodium meglumine+gentamicin 2,29	
Microspheres 6	358 (15.5)
Sodium tetradecyl sulfate foam 24,37	75 (3)
N-butyl-cyanocrylate	387 (17)
+iodized oil 23,24,41	
Absolute ethanol 23,29,32,43	302 (13)
Cyanoacrylate glue+iodized oil 25	29 (1)
PVA+coils 9,25,26,28,36,38,44,45,48	334 (14)
Isobutyl-2-cyanoacrylate glue+iodized oil 16,33	213 (9)
Gelatin sponge+iodized oil 42	14 (0.6)
Enbucrilate tissue adhesive+lipiodol 48	12 (0.5)
N-butyl-cyanocrylate+amplatzer vascular plug 44	45 (1.9)
Amplatzer vascular plug+coils 38	35 (1.5)
Glue+lipiodol+microparticles 27	62 (2.7)
Ethibloc+lipiodol 39	15 (0.6)
Trisacryl microspheres+gelform+coils 40	63 (2.7)
Histoacryl+lipiodol 17,28	48 (2)
Interval between PVE and surgery (day)	38.9
Resection post PVE	1782 (76)
No-resection post PVE	553 (24)

**Abbreviation:** HCC: hepatocellular carcinoma; CHC: cholangiocarcinoma; CLM: colorectal liver metastases; PVE: portal vein embolization.

**Table 4:** Complications related to PVE

Details	No.
Total no. patients	247
Abdominal pain 16,17,23,25,27	69
Fever 16,23,25,27,33,36,45	81
Coil displacement 6,9,23,29,33,37,40,41	42
Portal vein thrombosis 6,9,23,29,36,41,43,48	30
Subcapsular hematoma 6,9,29,32,33,36,39	14
Nausea and vomiting 33,44,45	12
Hepatic abscess 23,43,44	7
Subcapsular biloma 38,41	5
Esophageal bleeding 6,9	2
Liver failure 23	4
Hemoperitoneum 33,45	2
Portal hypertension 40	4
Systemic sepsis 33	1
Bile duct infection 42	1
Pseudoaneurysm 43	1
Pulmonary embolism 33	1
Intrahepatic portovenous shunt 40	1
Hepatic artery branch laceration 45	1
Bile leak 29	2
Bowel obstruction 29	1
Hyperbilirubinemia 44	1
Idiopathic hepatic venous thrombosis 44	1

## Abbreviations

PVE: portal vein embolization
FLR: future liver remnant
PVL: portal vein ligation
SIRT: selective internal radiation therapy
ALPPS: associating liver partition with portal vein ligation for staged hepatectomy
HCC: hepatocellular carcinoma
NOS: Newcastle-Ottawa Quality Assessment Scale
HGF: hepatic growth factor
TGF: transforming growth factor
GSA: 99Tc-galactosyl-human serum albumin
TIPE: transileocolic portal vein embolization
PTPE: percutaneous transhepatic portal vein embolization
PVA: polyvinyl alcohol
TACE: transarterial chemoembolization
ICG: indocyanine green
